# Supplementary figures and images for: LINC01936 inhibits the proliferation and metastasis of lung squamous cell carcinoma probably by EMT signaling and immune infiltration
Source: PeerJ. 2023 Dec 7;11:e16447. doi: 10.7717/peerj.16447 (PMC10710776; doi:10.7717/peerj.16447)

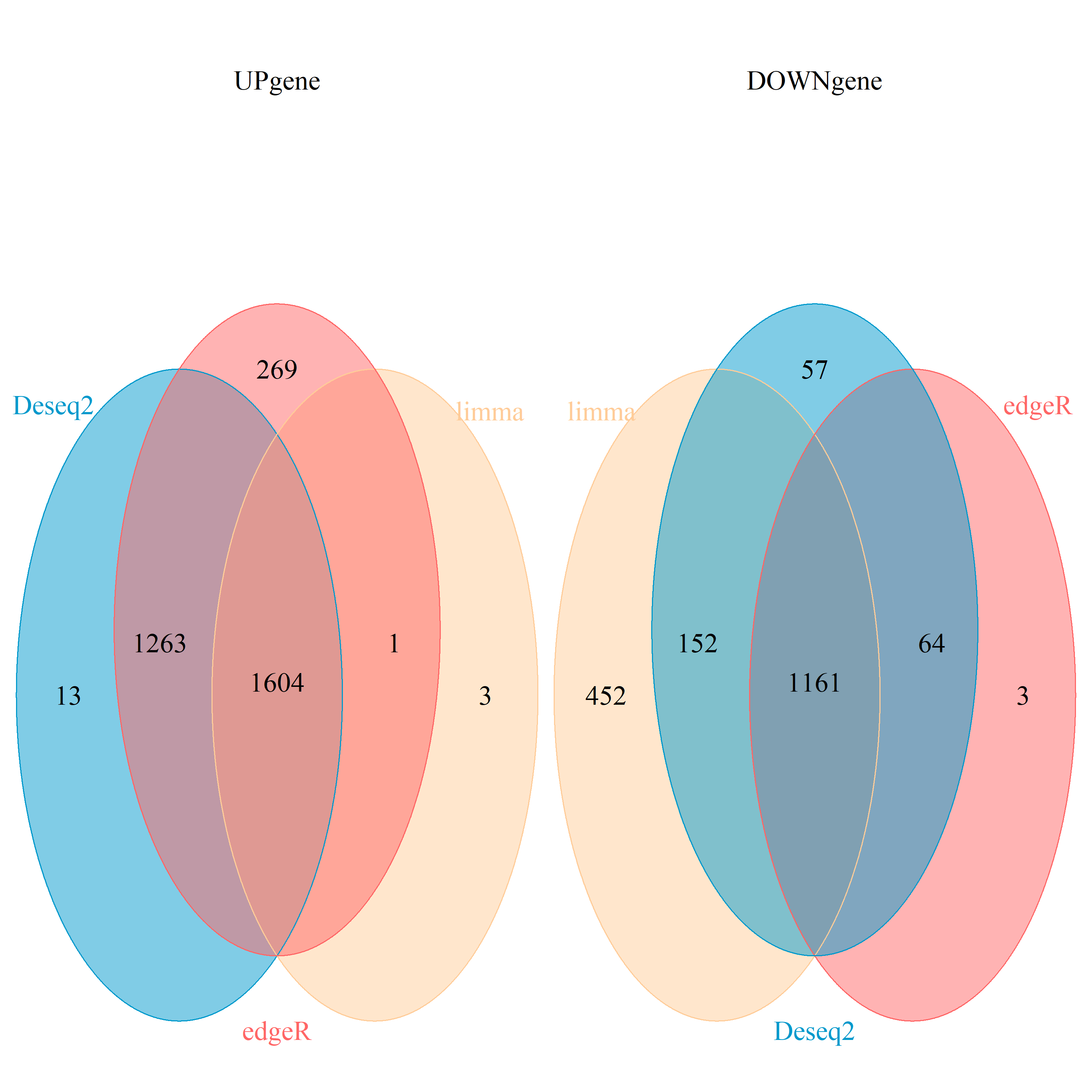

Supplement: Supplemental Information 1 — The differentially expressed lncRNAs from LUSC and normal tissues were collected using the DEseq2, edgeR and Limma packages in R platform. [file peerj-11-16447-s001.zip › S1.TCGA differentially expressed genes/01.TCGA-LUSC-lncRNA/deg.png]

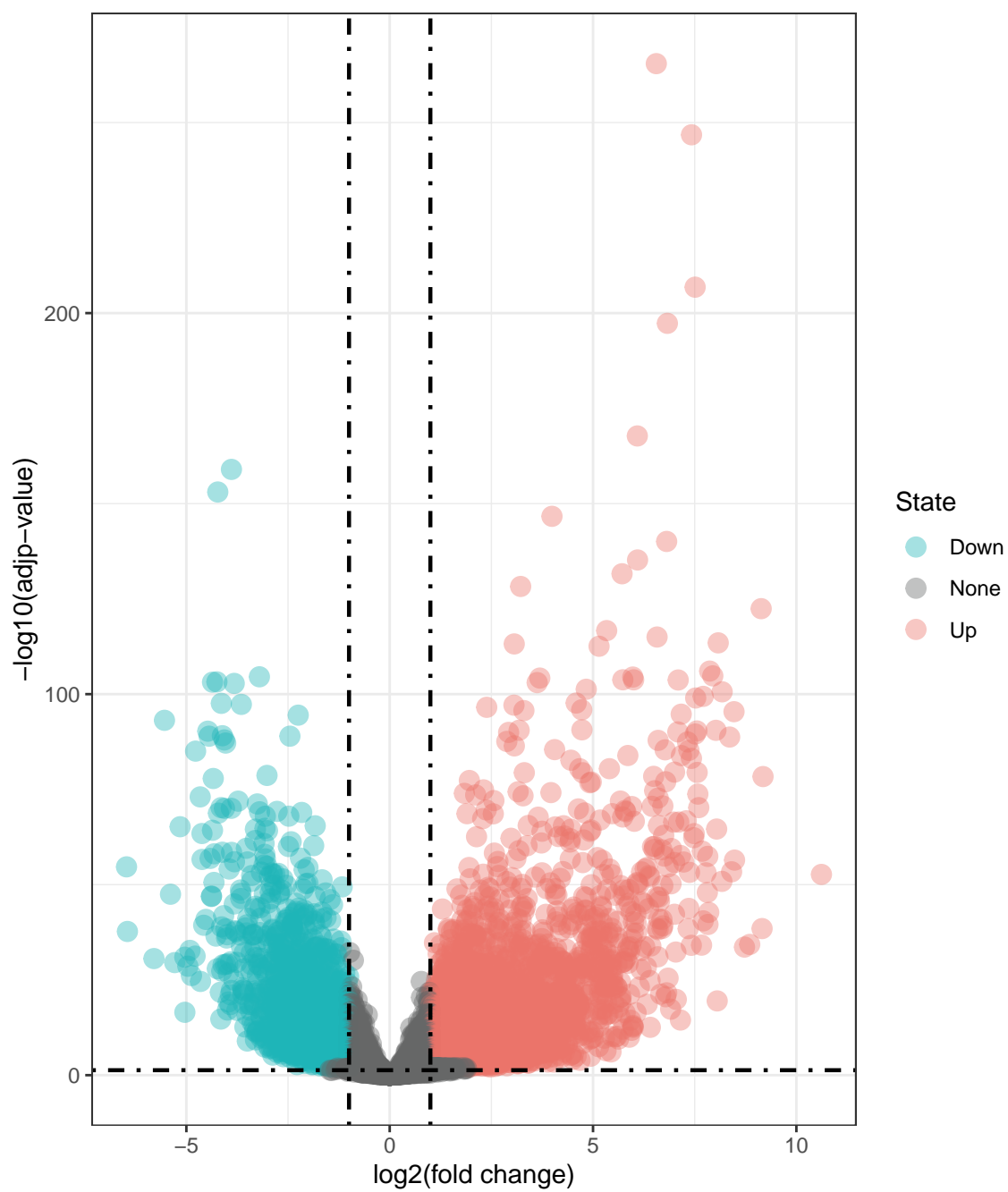

Supplement: Supplemental Information 1 — The differentially expressed lncRNAs from LUSC and normal tissues were collected using the DEseq2, edgeR and Limma packages in R platform. [file peerj-11-16447-s001.zip › S1.TCGA differentially expressed genes/02.Three Volcano map/DEseq2-vol/DEseq2-vol/DESeq2_volcano.pdf]

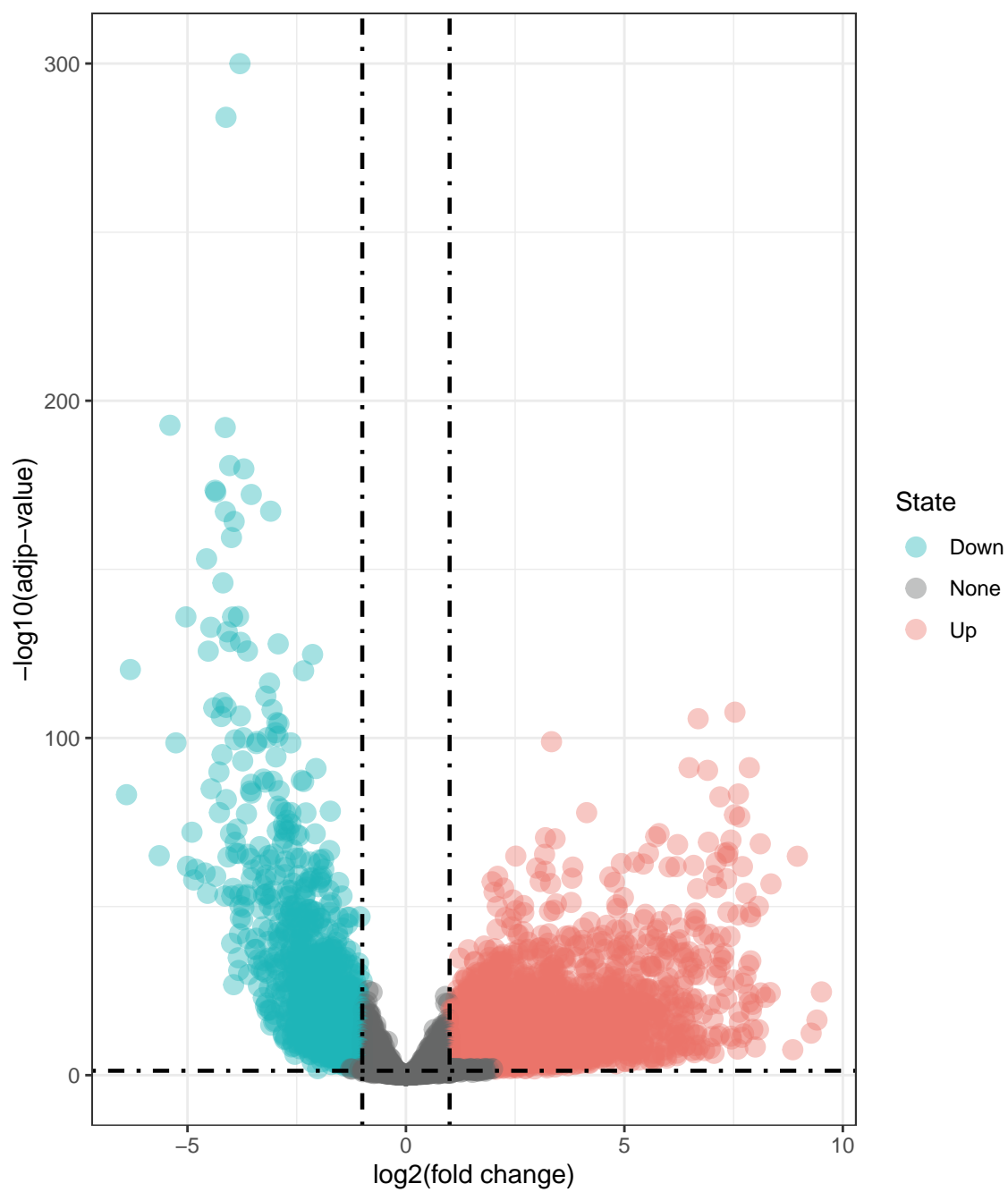

Supplement: Supplemental Information 1 — The differentially expressed lncRNAs from LUSC and normal tissues were collected using the DEseq2, edgeR and Limma packages in R platform. [file peerj-11-16447-s001.zip › S1.TCGA differentially expressed genes/02.Three Volcano map/edger-vol/edger-vol/edgeR_volcano.pdf]

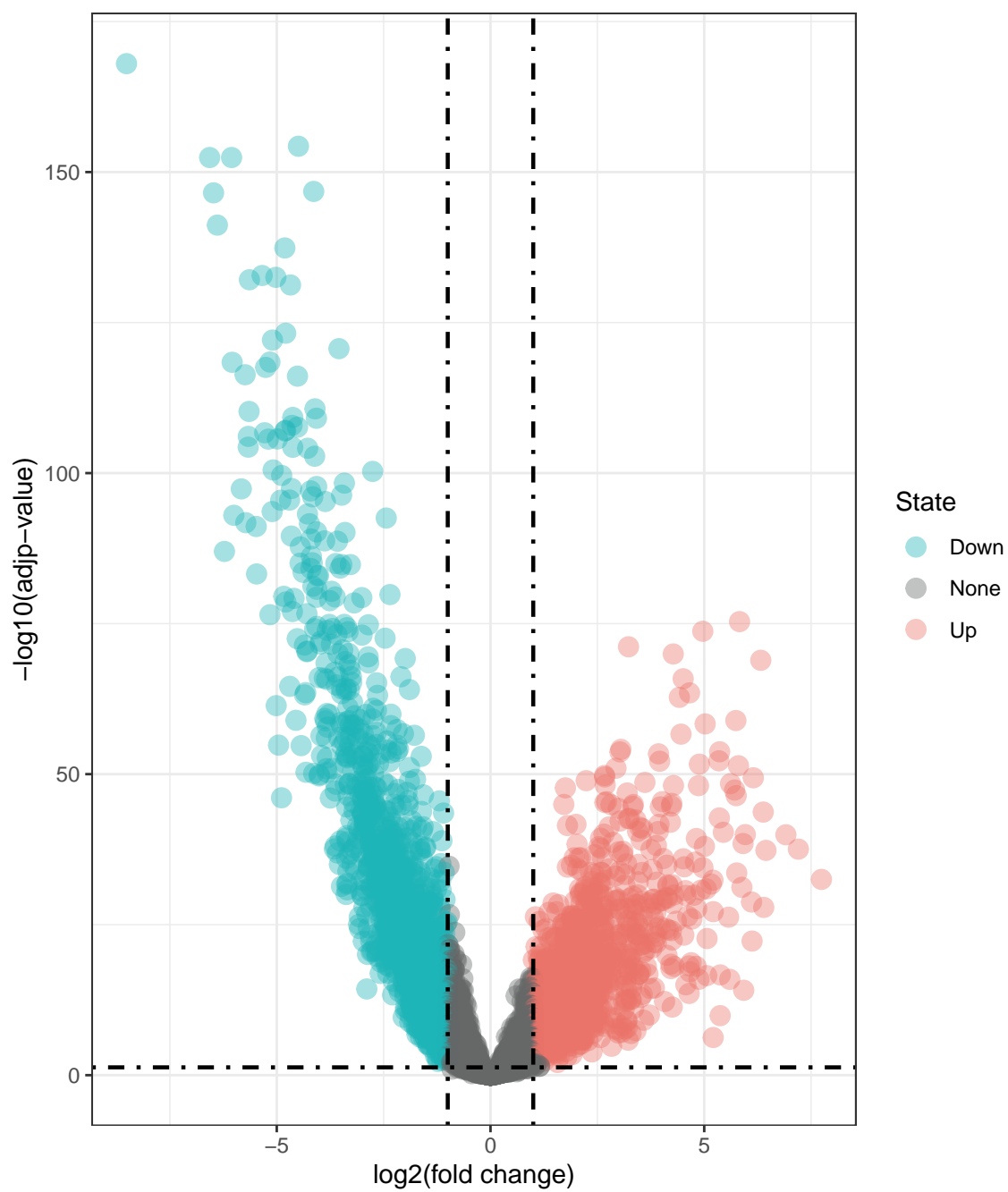

Supplement: Supplemental Information 1 — The differentially expressed lncRNAs from LUSC and normal tissues were collected using the DEseq2, edgeR and Limma packages in R platform. [file peerj-11-16447-s001.zip › S1.TCGA differentially expressed genes/02.Three Volcano map/limma-vol/limma-vol/limma_volcano.pdf]

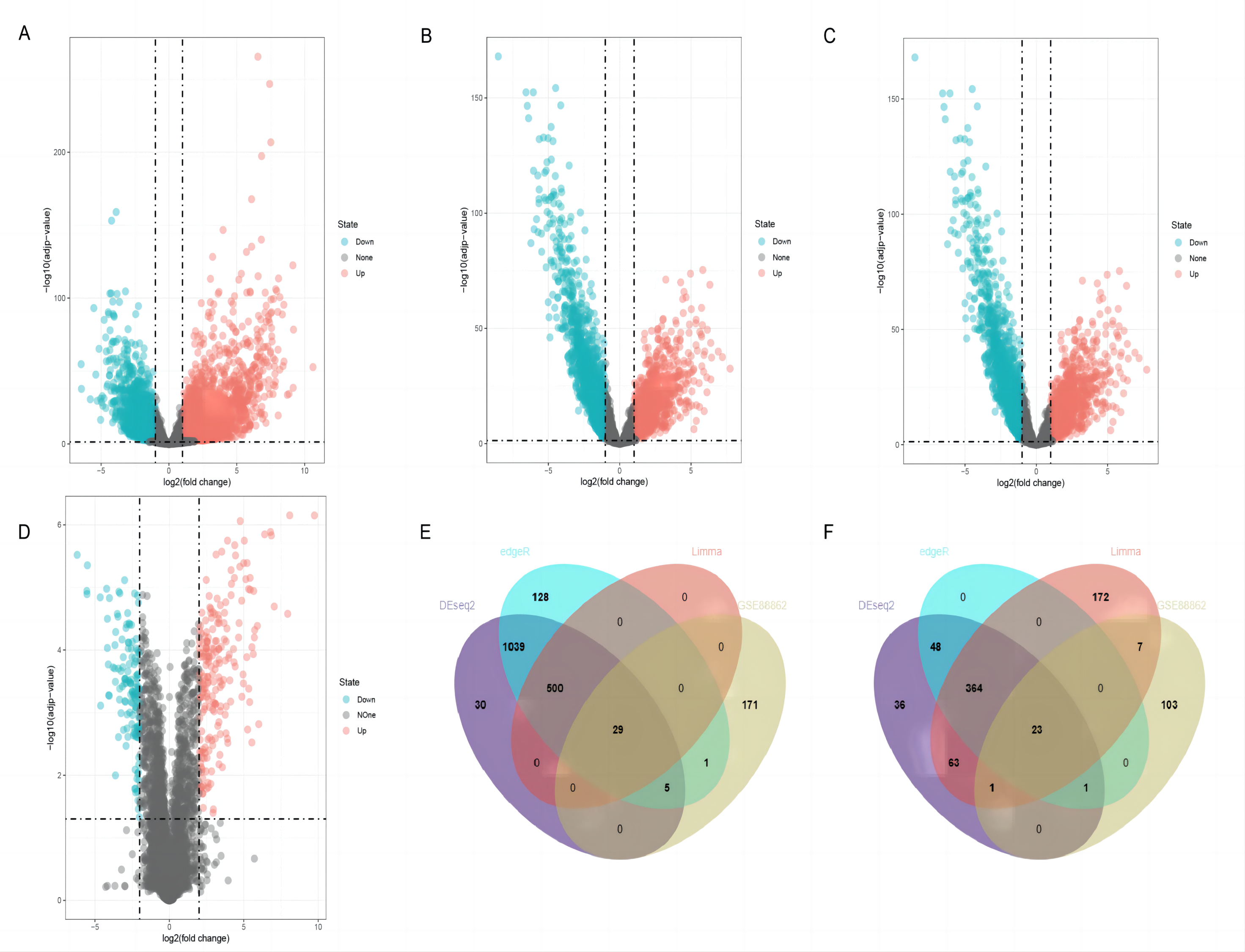

Supplement: Supplemental Information 1 — The differentially expressed lncRNAs from LUSC and normal tissues were collected using the DEseq2, edgeR and Limma packages in R platform. [file peerj-11-16447-s001.zip › S1.TCGA differentially expressed genes/figure2.png]

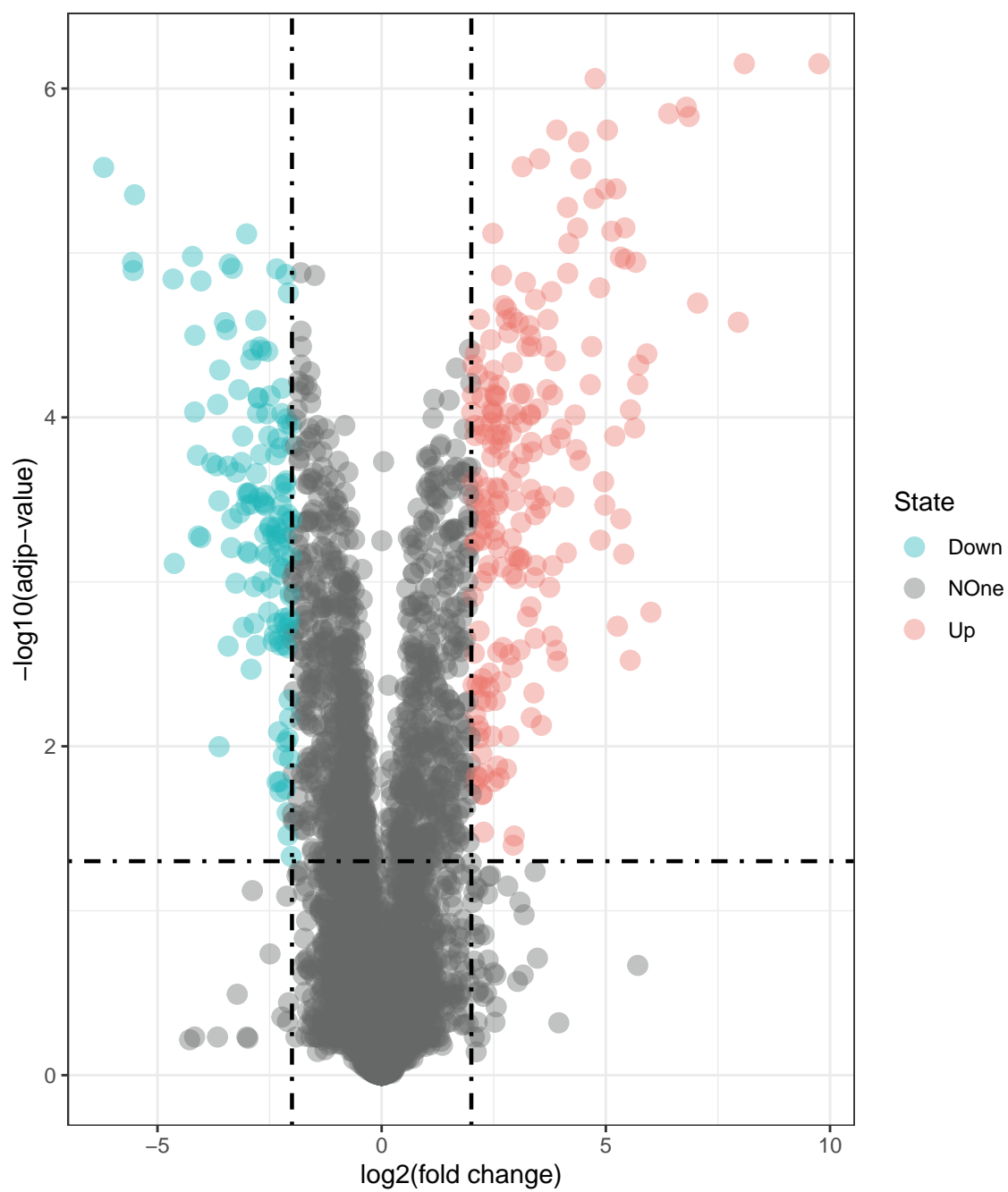

Supplement: Supplemental Information 2 — The differentially expressed lncRNAs from GSE88862 was identified by the online analytic tool GEO2R provided by the GEO database. [file peerj-11-16447-s002.zip › S2.GSE88862 differentially expressed genes/GSE88862_volcano.pdf]

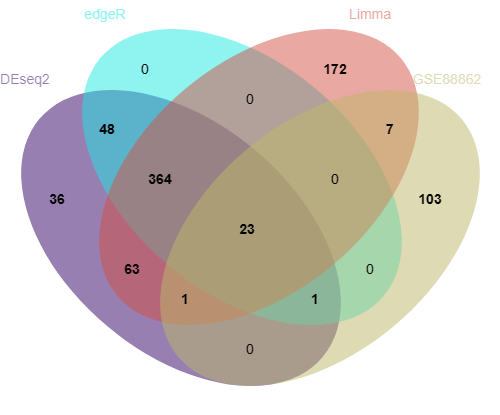

Supplement: Supplemental Information 3 — VENNY 2.1.0 was employed to draw Venn diagrams based on the differentially expressed lncRNAs. [file peerj-11-16447-s003.zip › S3.TCGA_GSE88862_venn/TCGA-GSE88862-23down.png]

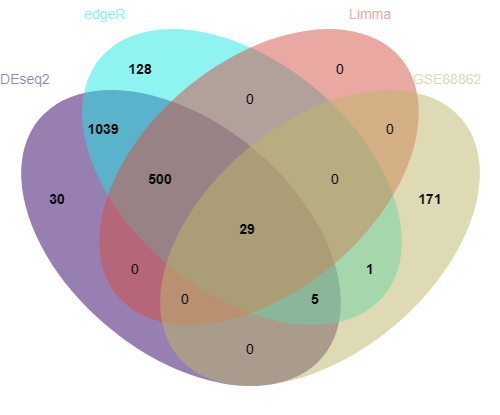

Supplement: Supplemental Information 3 — VENNY 2.1.0 was employed to draw Venn diagrams based on the differentially expressed lncRNAs. [file peerj-11-16447-s003.zip › S3.TCGA_GSE88862_venn/TCGA-GSE88862-29up.png]

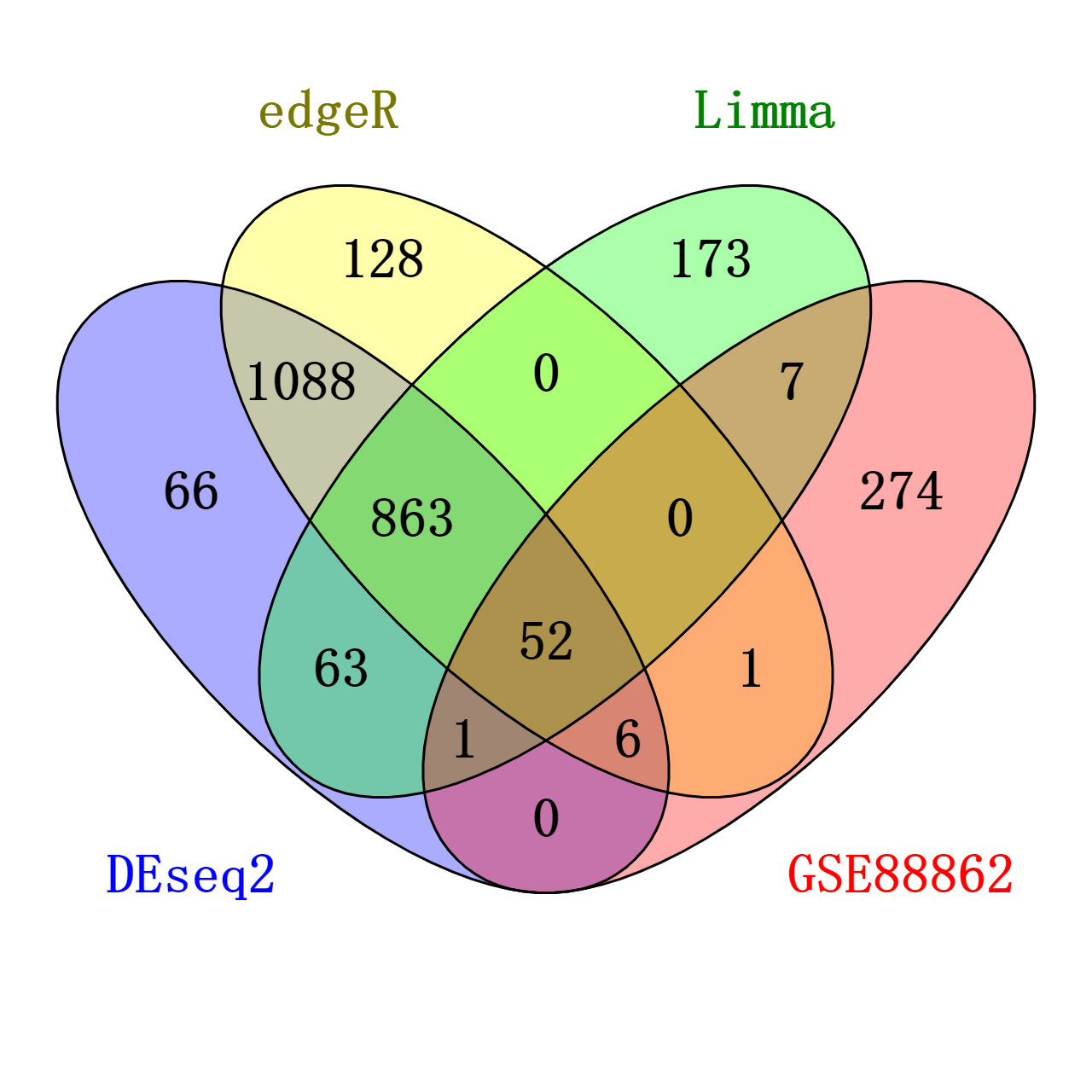

Supplement: Supplemental Information 3 — VENNY 2.1.0 was employed to draw Venn diagrams based on the differentially expressed lncRNAs. [file peerj-11-16447-s003.zip › S3.TCGA_GSE88862_venn/TCGA-GSE88862-52venn.png]

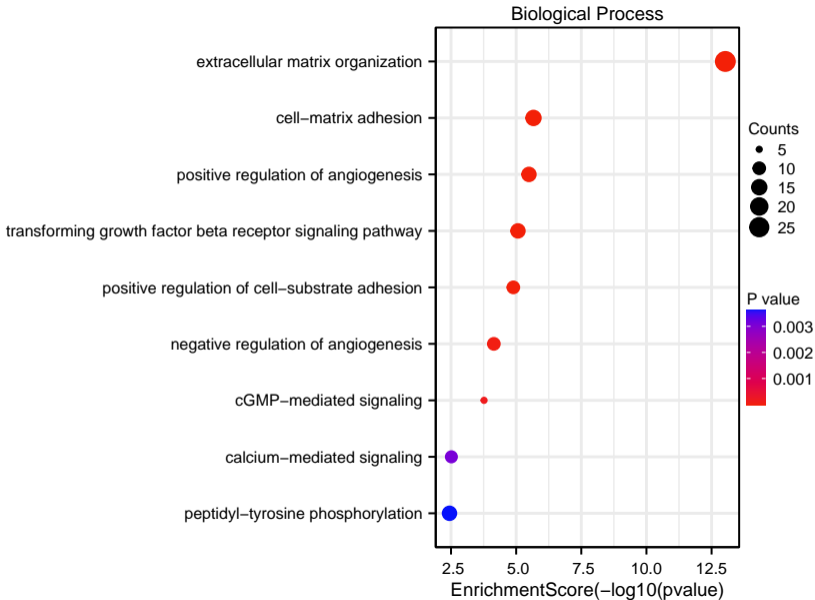

Supplement: Supplemental Information 5 — The ggplot2 R package and the DAVID 6.8 database were used to establish Gene Ontology (GO) and Kyoto Encyclopedia of Genes and Genomes (KEGG) analysis. The GSVA R package was used to study the pathways related to LINC01936. [file peerj-11-16447-s005.zip › S5. KEGG GO GSVA/BP.pdf]

## Cellular Component

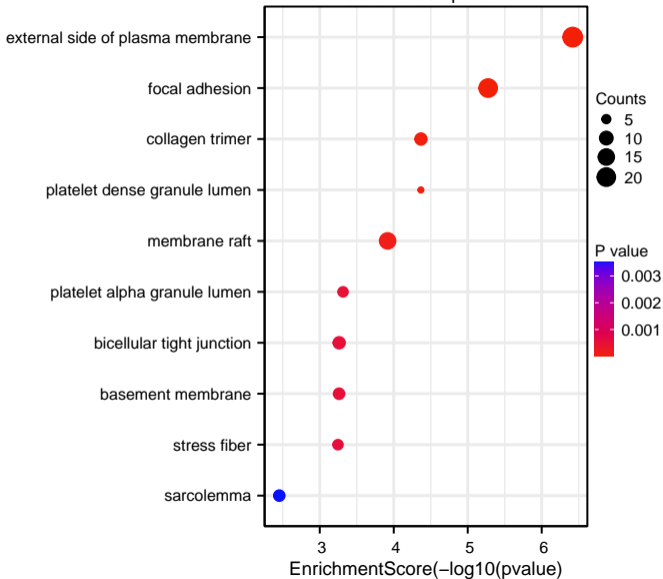

Supplement: Supplemental Information 5 — The ggplot2 R package and the DAVID 6.8 database were used to establish Gene Ontology (GO) and Kyoto Encyclopedia of Genes and Genomes (KEGG) analysis. The GSVA R package was used to study the pathways related to LINC01936. [file peerj-11-16447-s005.zip › S5. KEGG GO GSVA/CC.pdf]

A

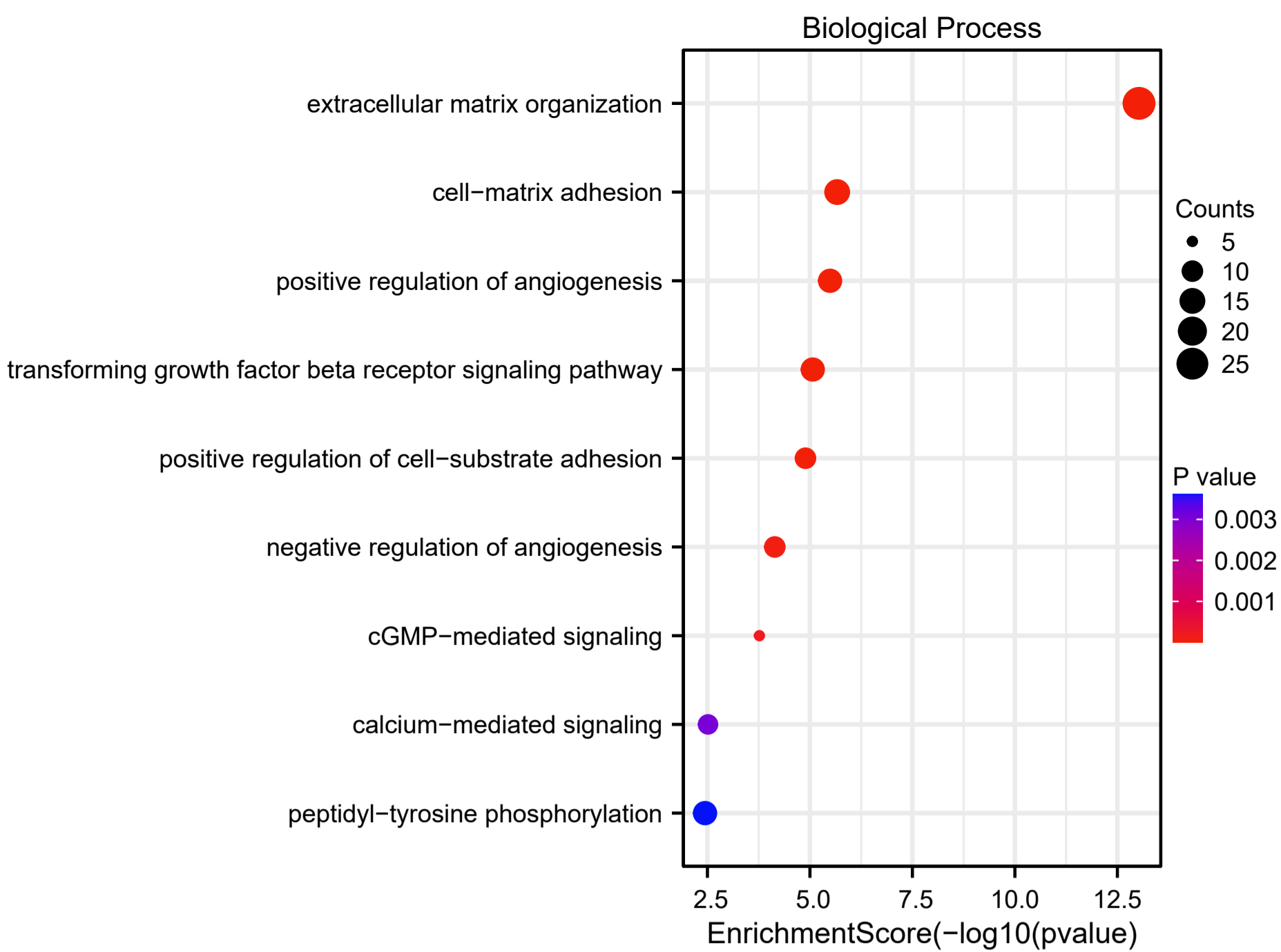

B

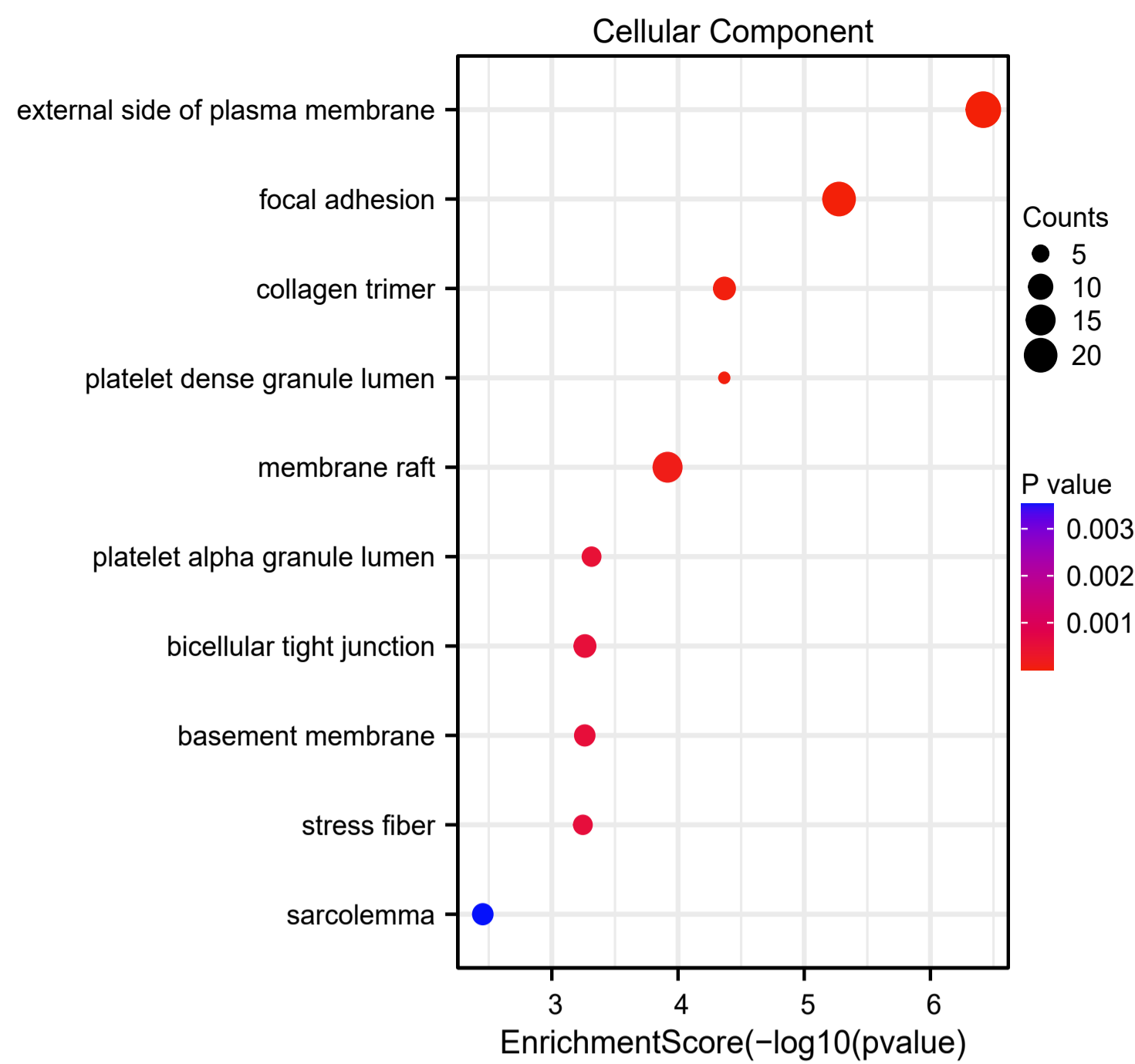

C

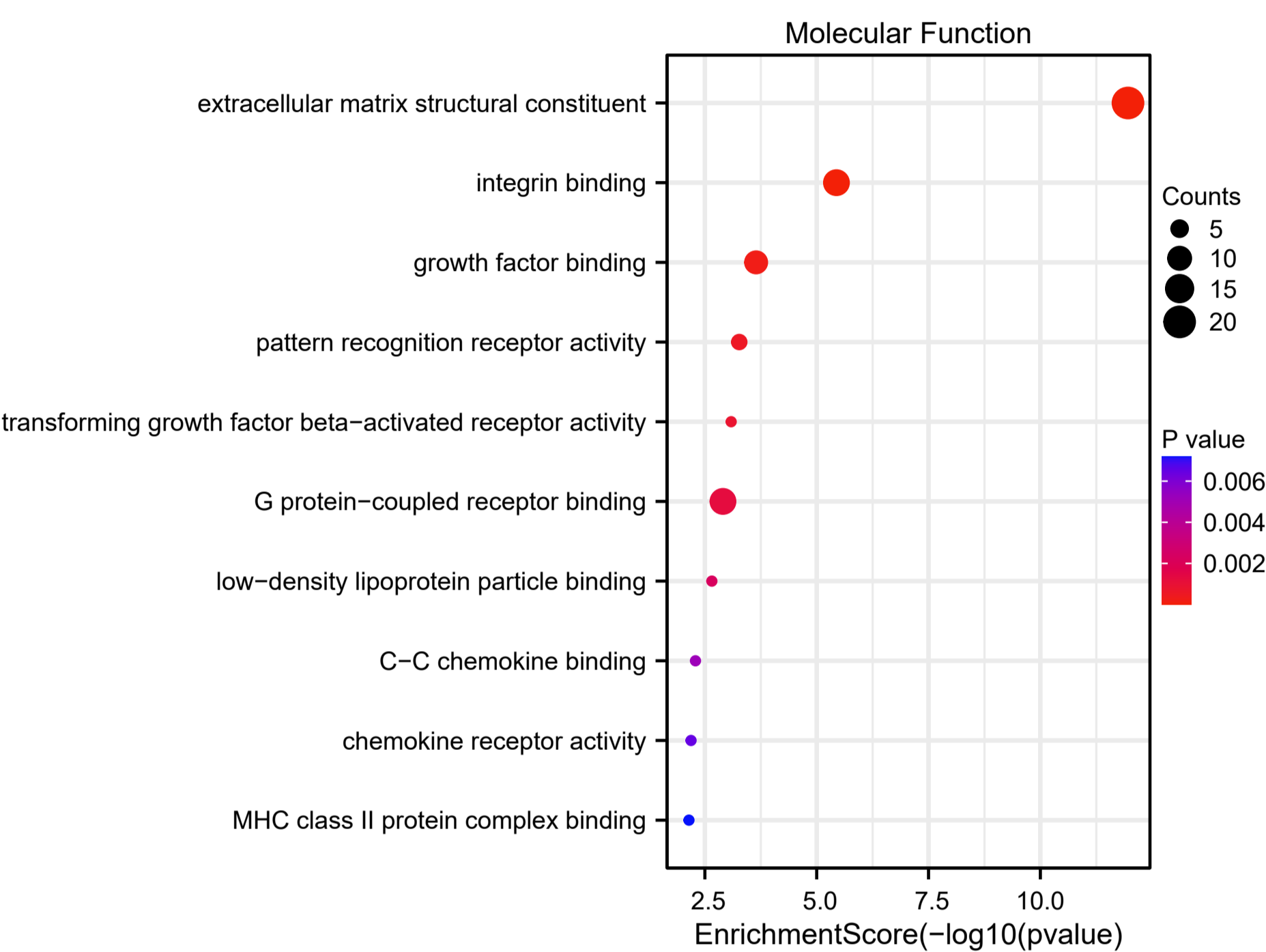

D

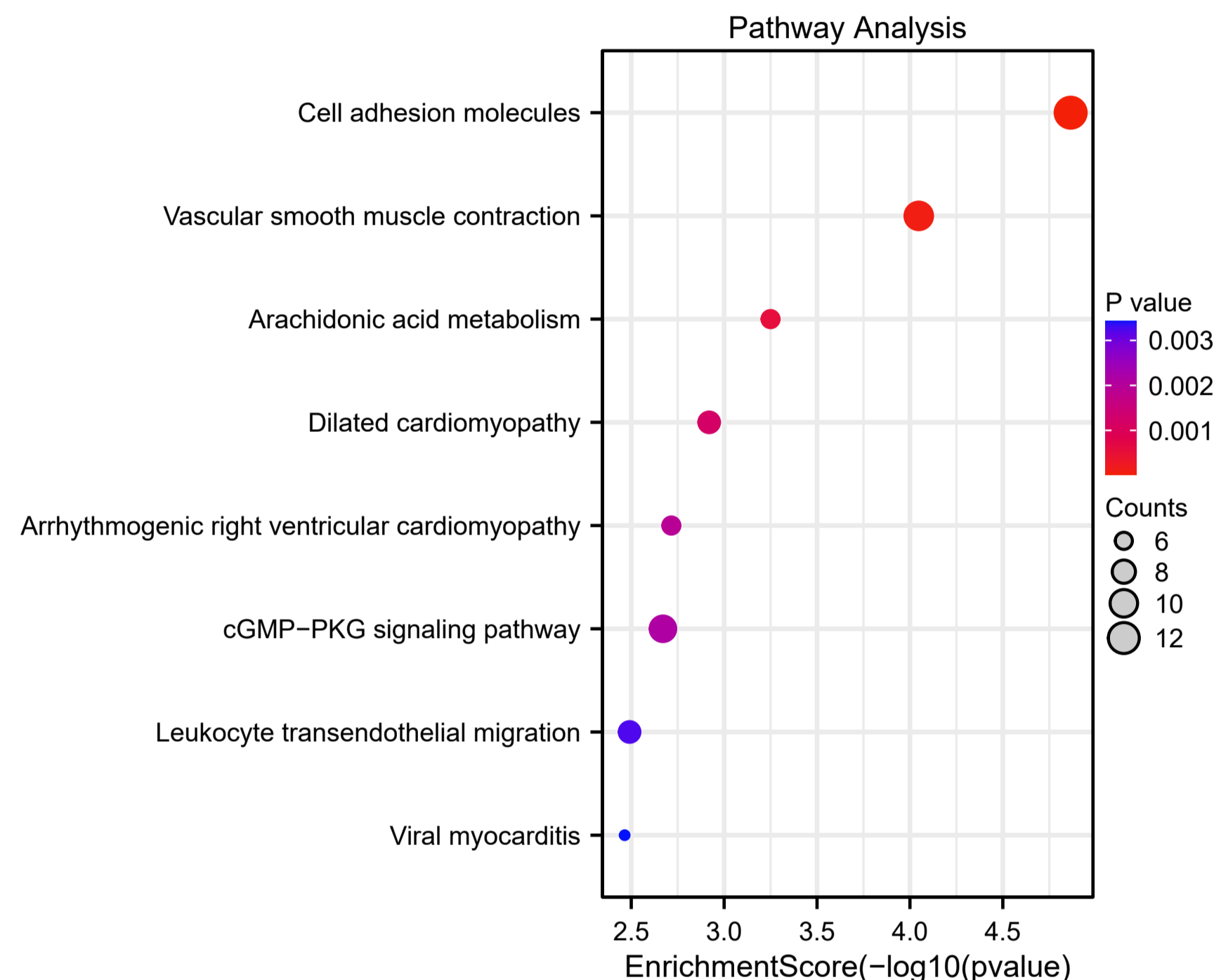

E

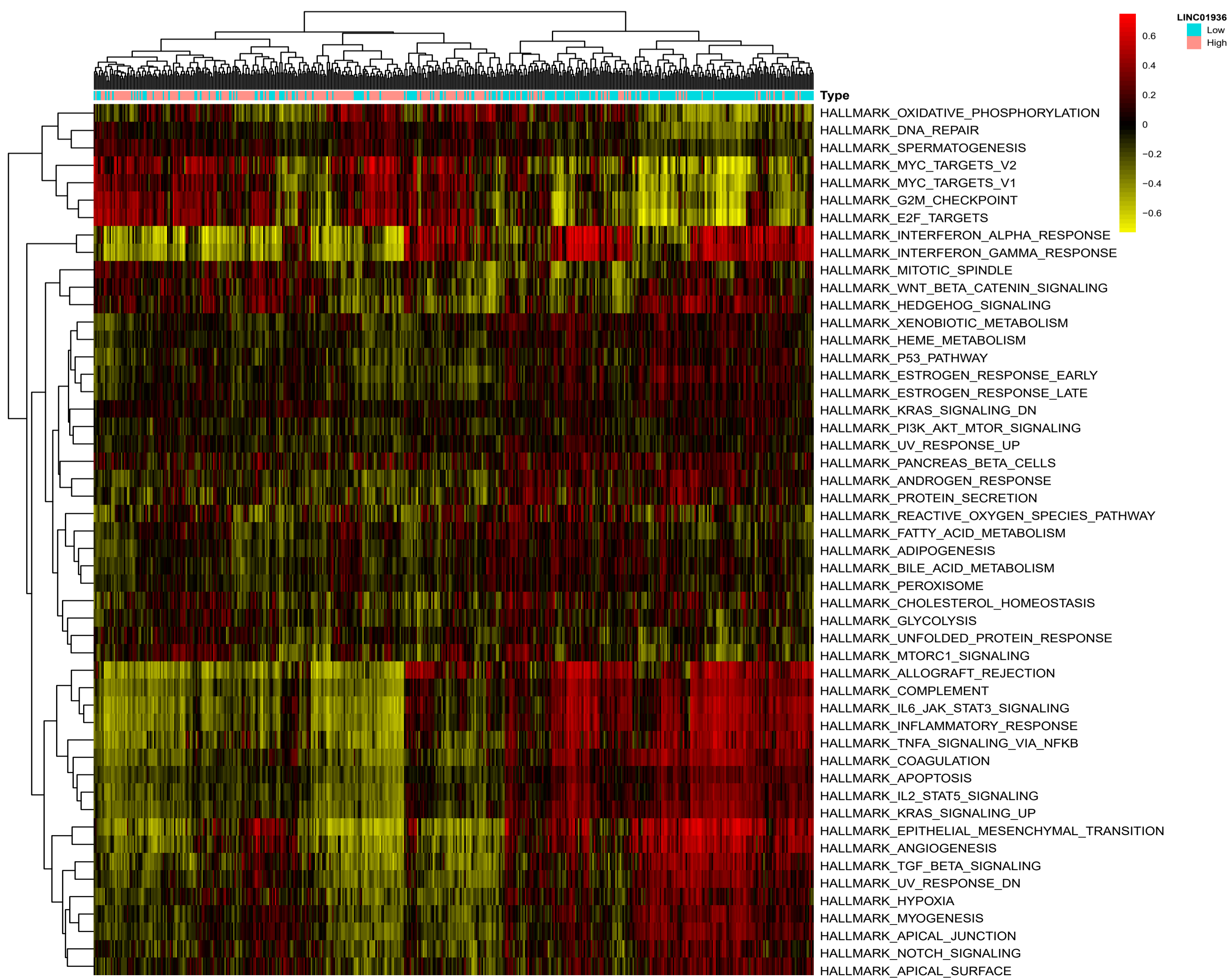

Supplement: Supplemental Information 5 — The ggplot2 R package and the DAVID 6.8 database were used to establish Gene Ontology (GO) and Kyoto Encyclopedia of Genes and Genomes (KEGG) analysis. The GSVA R package was used to study the pathways related to LINC01936. [file peerj-11-16447-s005.zip › S5. KEGG GO GSVA/GO KEGG GSVA.pdf]

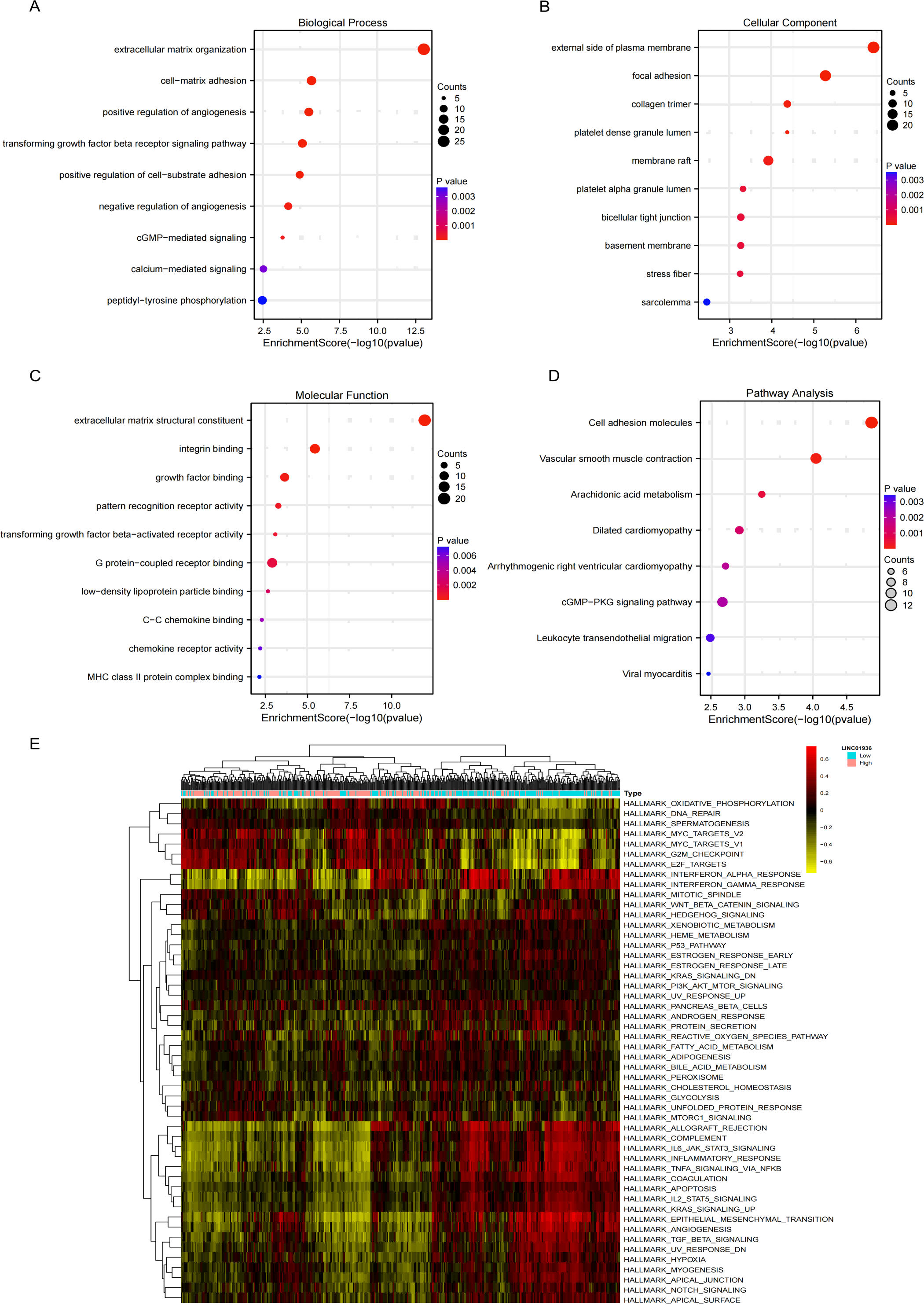

Supplement: Supplemental Information 5 — The ggplot2 R package and the DAVID 6.8 database were used to establish Gene Ontology (GO) and Kyoto Encyclopedia of Genes and Genomes (KEGG) analysis. The GSVA R package was used to study the pathways related to LINC01936. [file peerj-11-16447-s005.zip › S5. KEGG GO GSVA/GO KEGG GSVA_00.png]

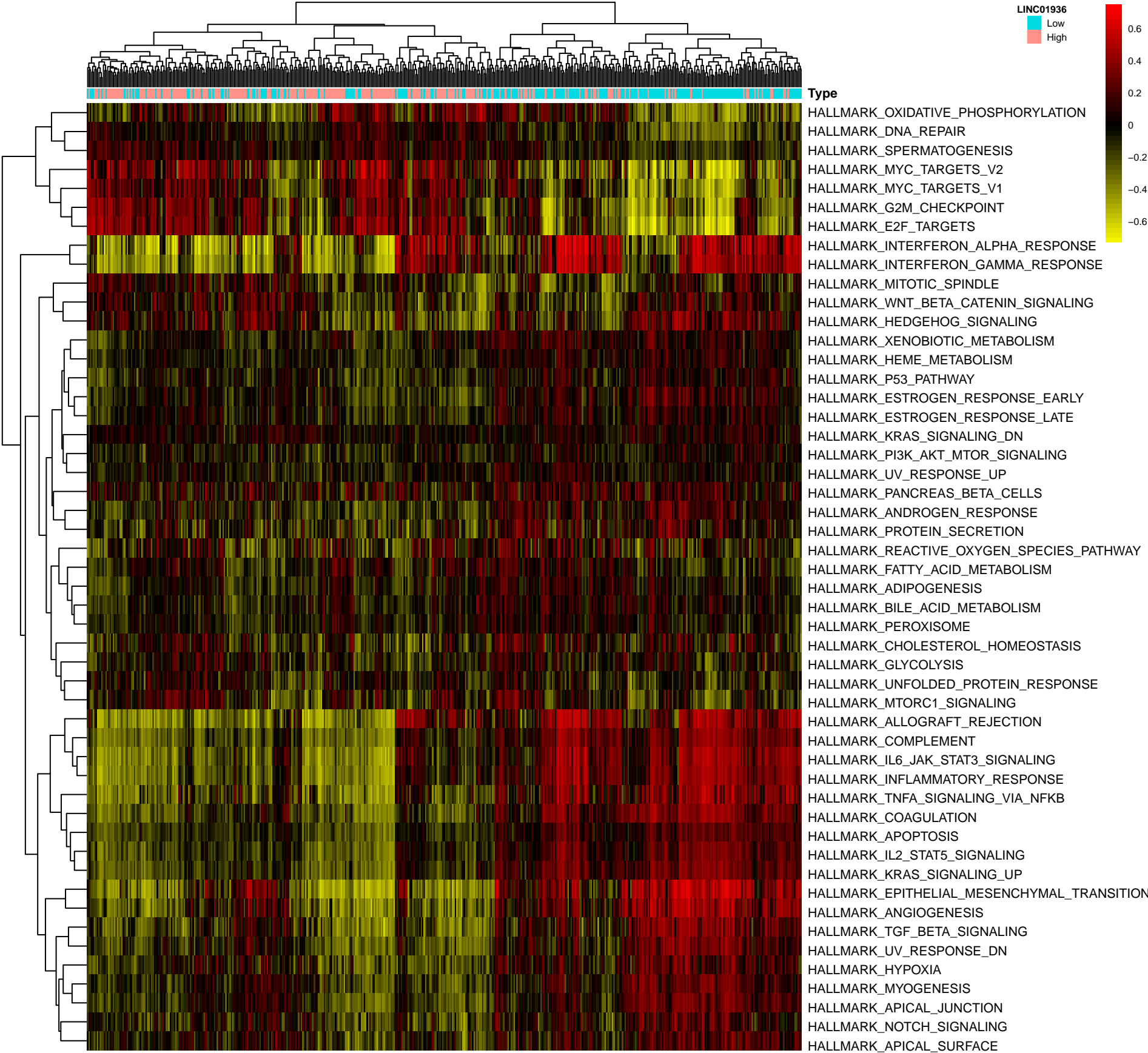

Supplement: Supplemental Information 5 — The ggplot2 R package and the DAVID 6.8 database were used to establish Gene Ontology (GO) and Kyoto Encyclopedia of Genes and Genomes (KEGG) analysis. The GSVA R package was used to study the pathways related to LINC01936. [file peerj-11-16447-s005.zip › S5. KEGG GO GSVA/GSVA.pdf]

## Pathway Analysis

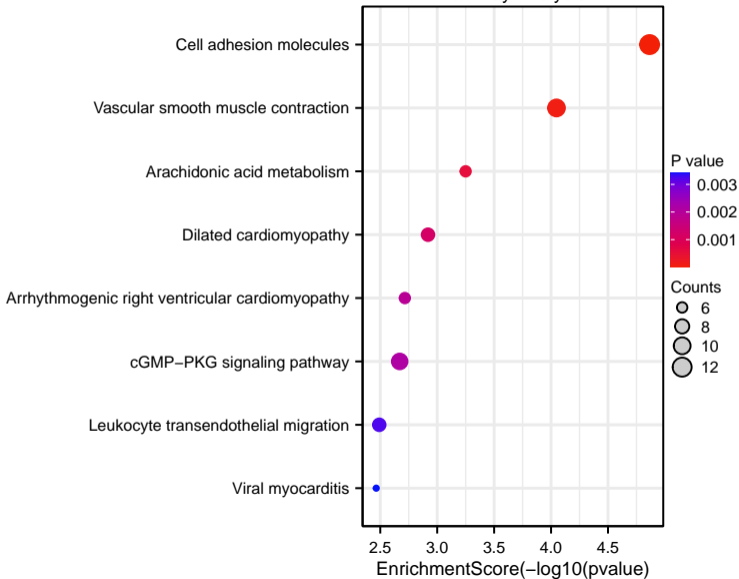

Supplement: Supplemental Information 5 — The ggplot2 R package and the DAVID 6.8 database were used to establish Gene Ontology (GO) and Kyoto Encyclopedia of Genes and Genomes (KEGG) analysis. The GSVA R package was used to study the pathways related to LINC01936. [file peerj-11-16447-s005.zip › S5. KEGG GO GSVA/KEGG.pdf]

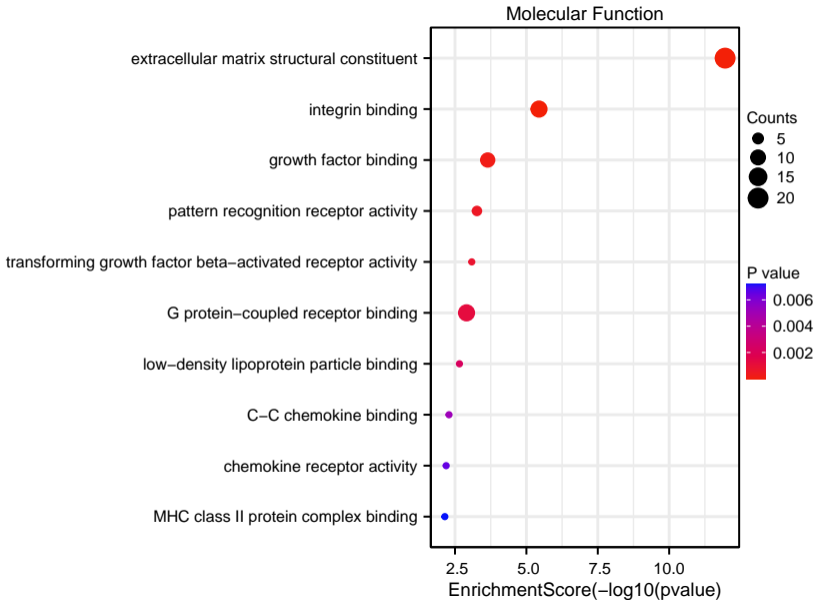

Supplement: Supplemental Information 5 — The ggplot2 R package and the DAVID 6.8 database were used to establish Gene Ontology (GO) and Kyoto Encyclopedia of Genes and Genomes (KEGG) analysis. The GSVA R package was used to study the pathways related to LINC01936. [file peerj-11-16447-s005.zip › S5. KEGG GO GSVA/MF.pdf]

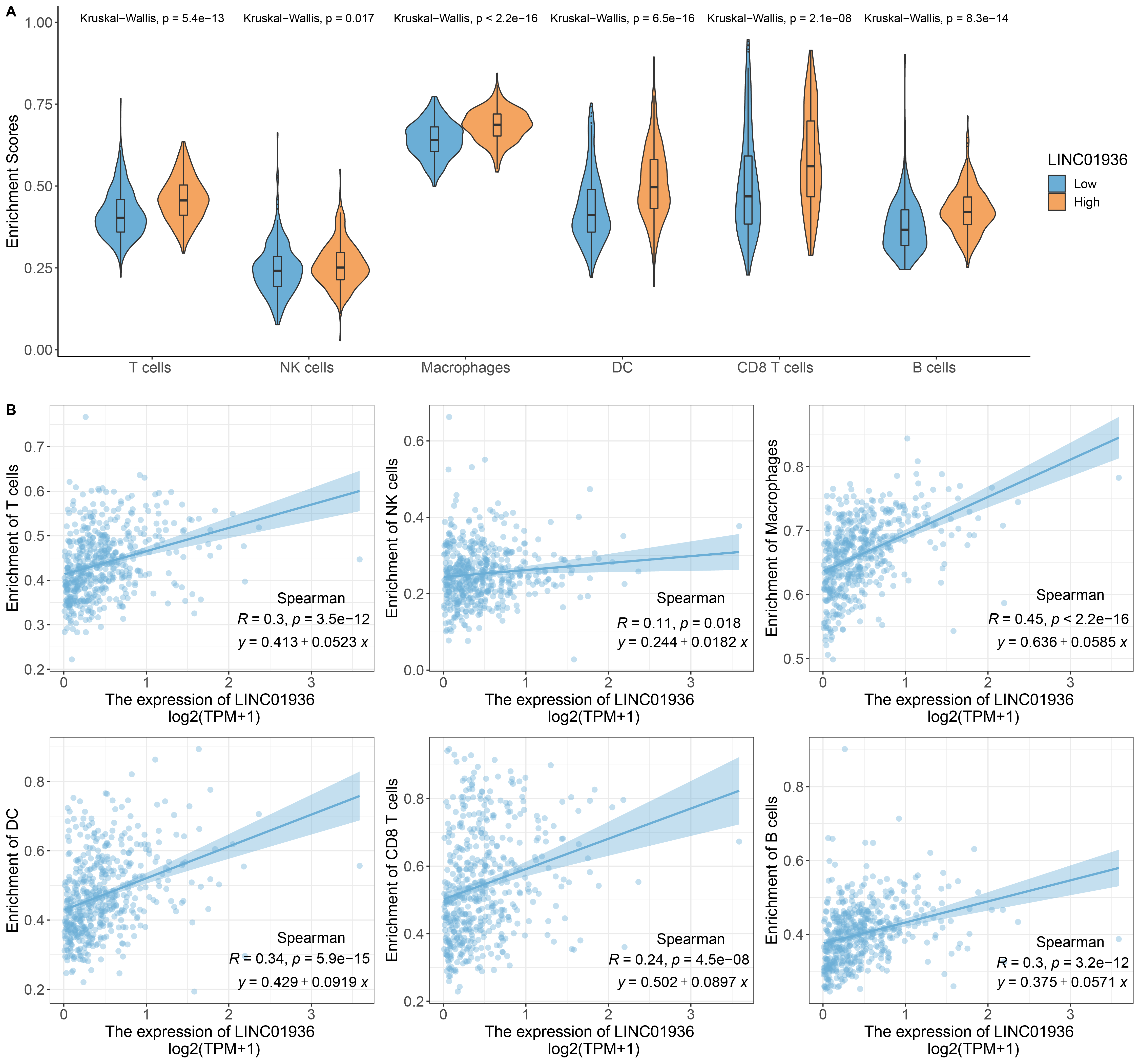

Supplement: Supplemental Information 7 — Pearson Correlation Analysis with ggpubr R package was used for analysis of tumor-infiltrating immune cells, and the correlations between the infiltrating level of immune cells. [file peerj-11-16447-s007.zip › S7. Immune infiltration/Immune score violins catter.tif]

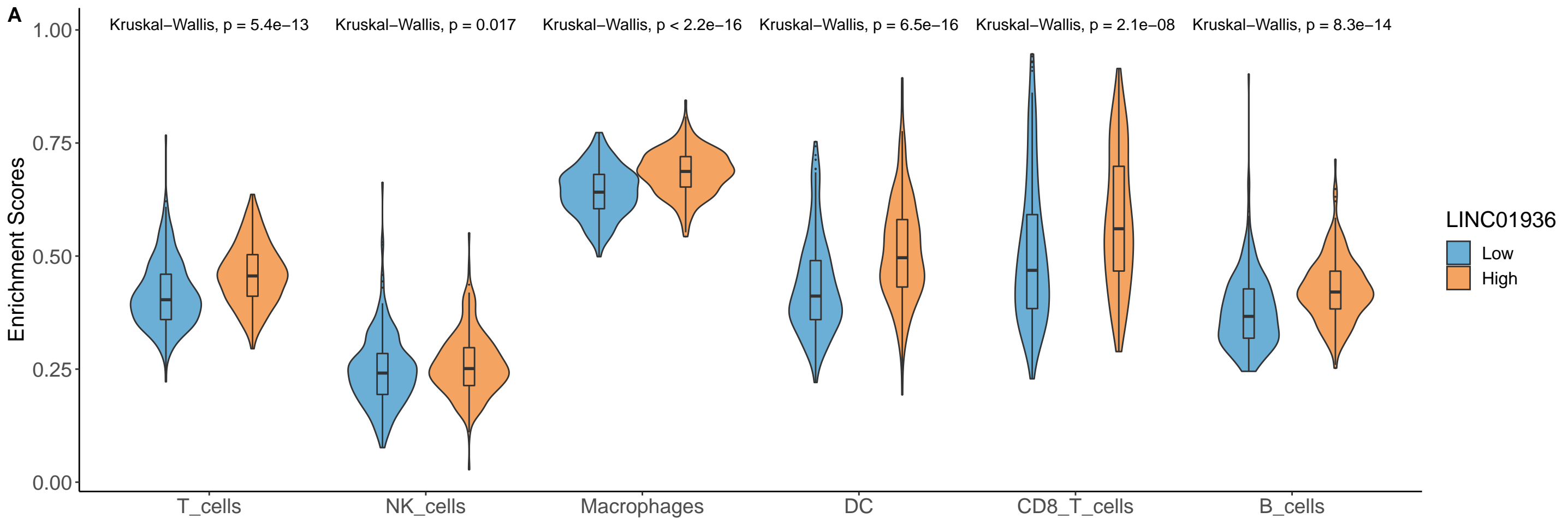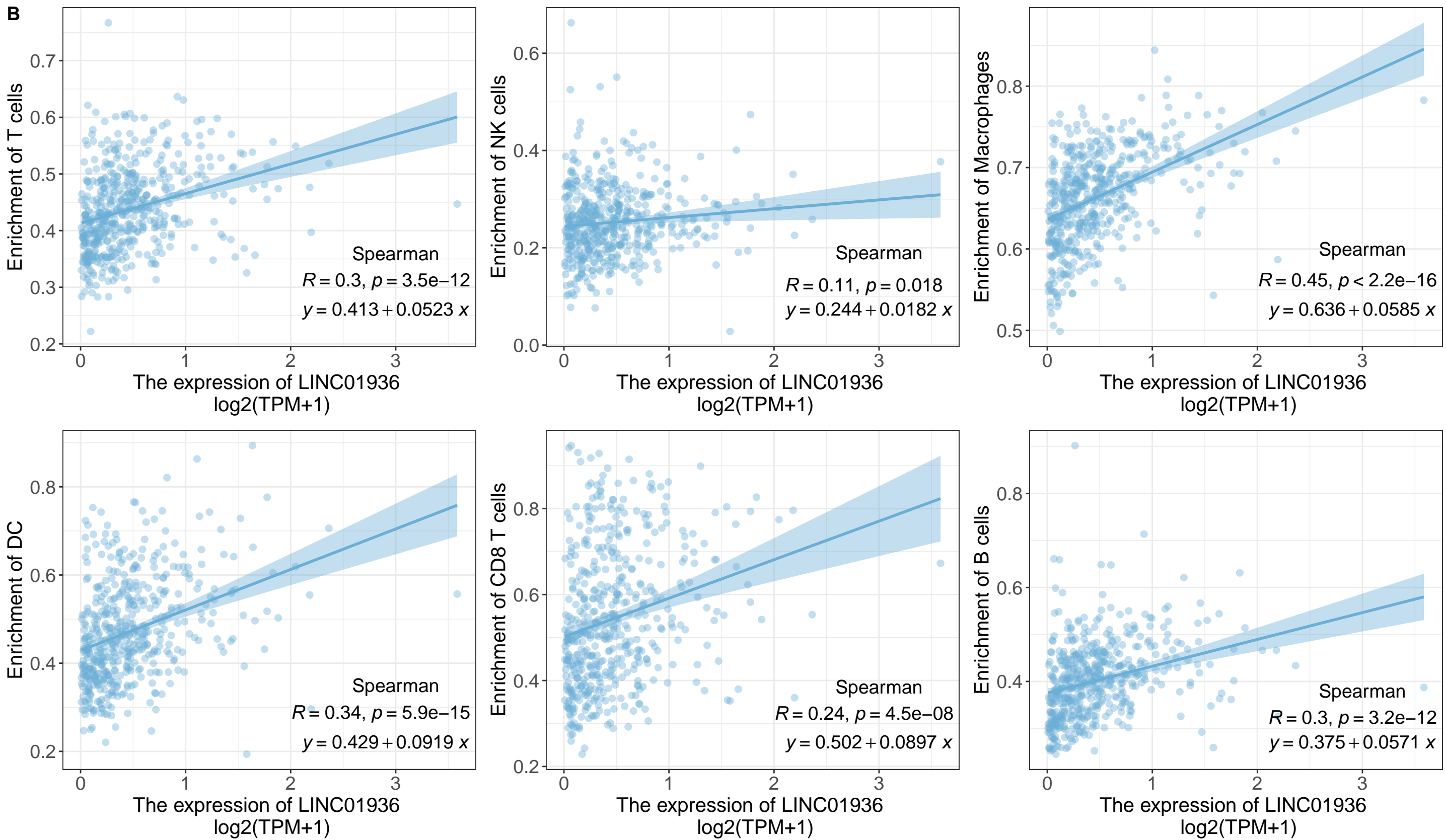

Supplement: Supplemental Information 7 — Pearson Correlation Analysis with ggpubr R package was used for analysis of tumor-infiltrating immune cells, and the correlations between the infiltrating level of immune cells. [file peerj-11-16447-s007.zip › S7. Immune infiltration/Immune_score_violin _scatter.pdf]

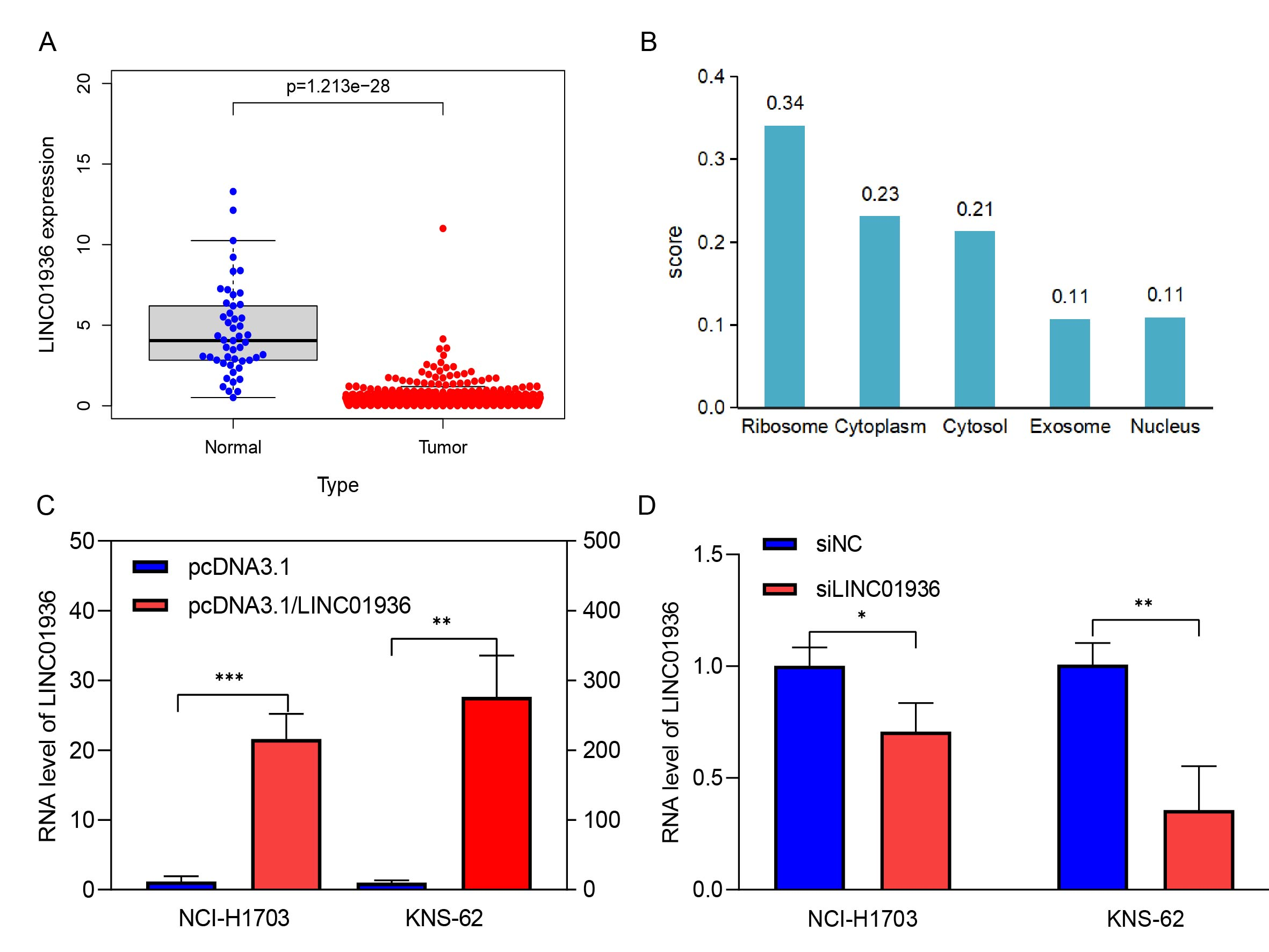

Supplement: Supplemental Information 8 — LINC01936 expression level was measured using the ABIPRISM® 7900HT Fast Real-Time PCR and reaction condition was conducted utilizing the Power SYBR Green PCR Master Mix. [file peerj-11-16447-s008.zip › S8. RT-PCR/figure3.png]

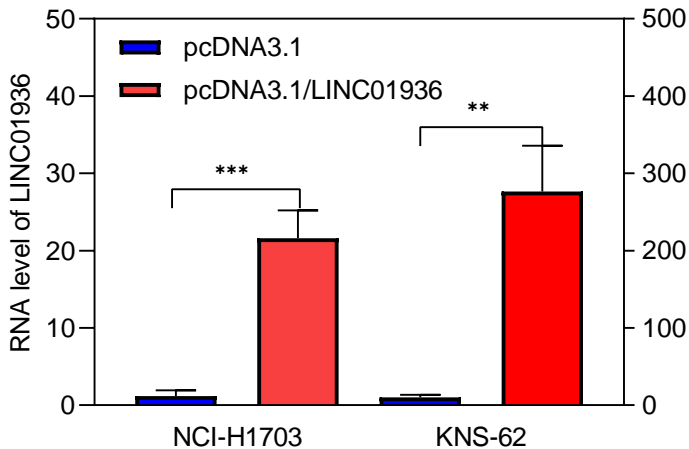

Supplement: Supplemental Information 8 — LINC01936 expression level was measured using the ABIPRISM® 7900HT Fast Real-Time PCR and reaction condition was conducted utilizing the Power SYBR Green PCR Master Mix. [file peerj-11-16447-s008.zip › S8. RT-PCR/qRT-PCR OV-LINC01936 in H1703 KNS62.pdf]

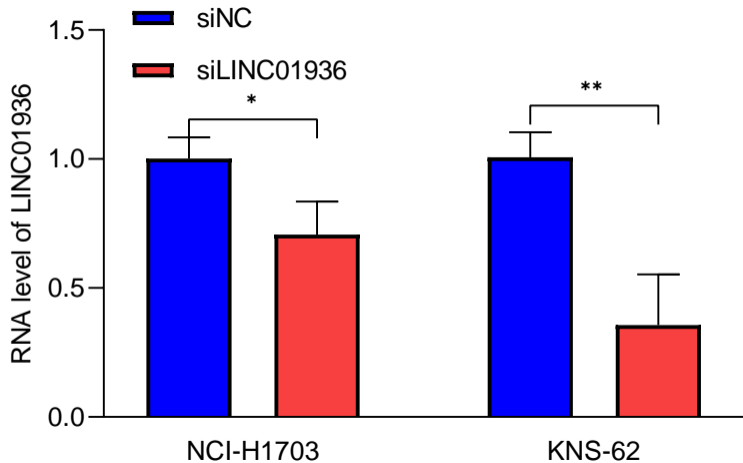

Supplement: Supplemental Information 8 — LINC01936 expression level was measured using the ABIPRISM® 7900HT Fast Real-Time PCR and reaction condition was conducted utilizing the Power SYBR Green PCR Master Mix. [file peerj-11-16447-s008.zip › S8. RT-PCR/qRT-PCR si-LINC01936 in H1703 KNS62.pdf]

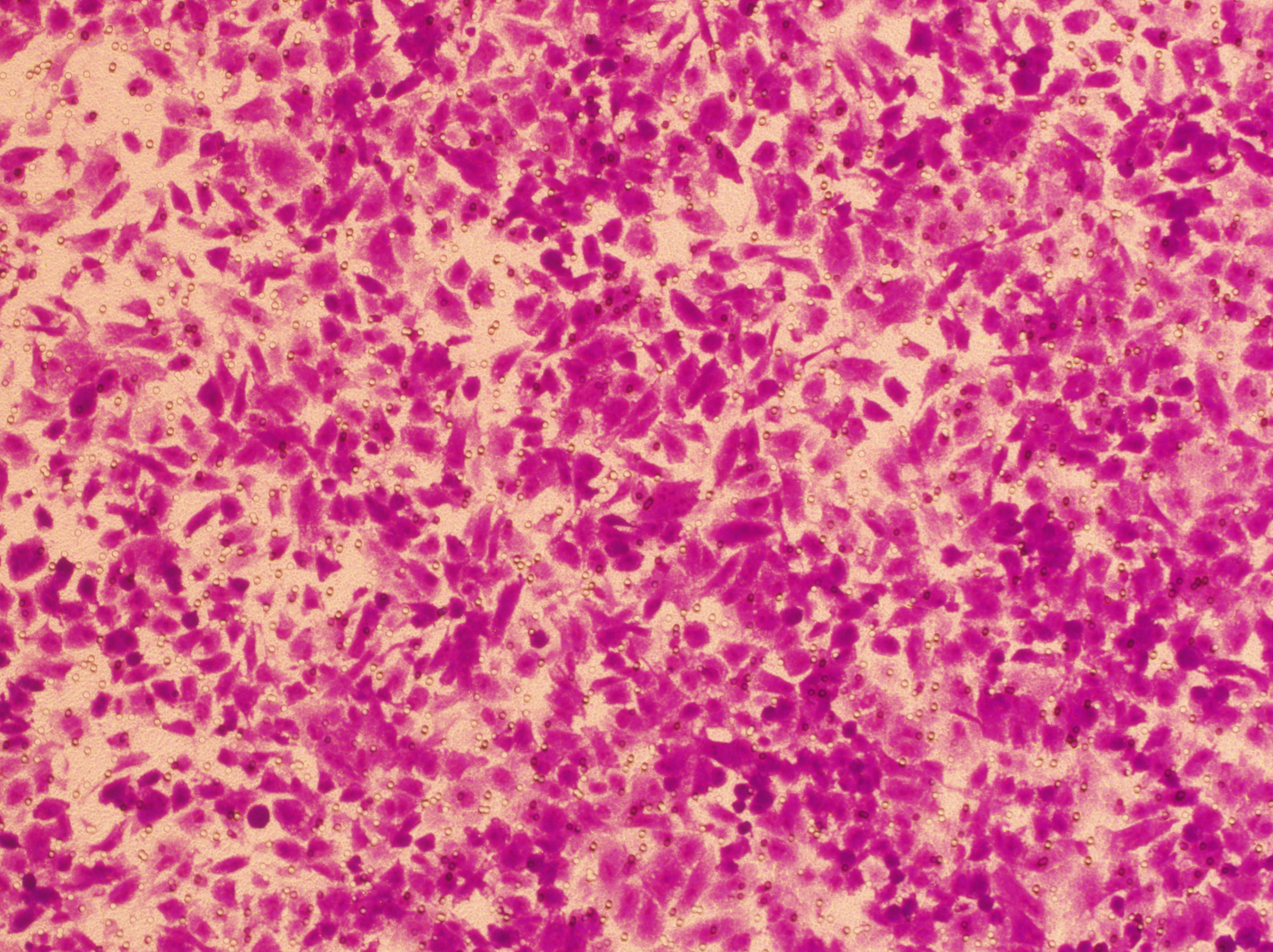

Supplement: Supplemental Information 12 — Migrated cells after silencing LINC01936 were photographed under IX71 inverted fluorescence microscope (magnification, ×100) [file peerj-11-16447-s012.zip › S11.2 Migration Si-LINC01936/H1703-siRNA/Migration siLINC01936 H1703.jpg]

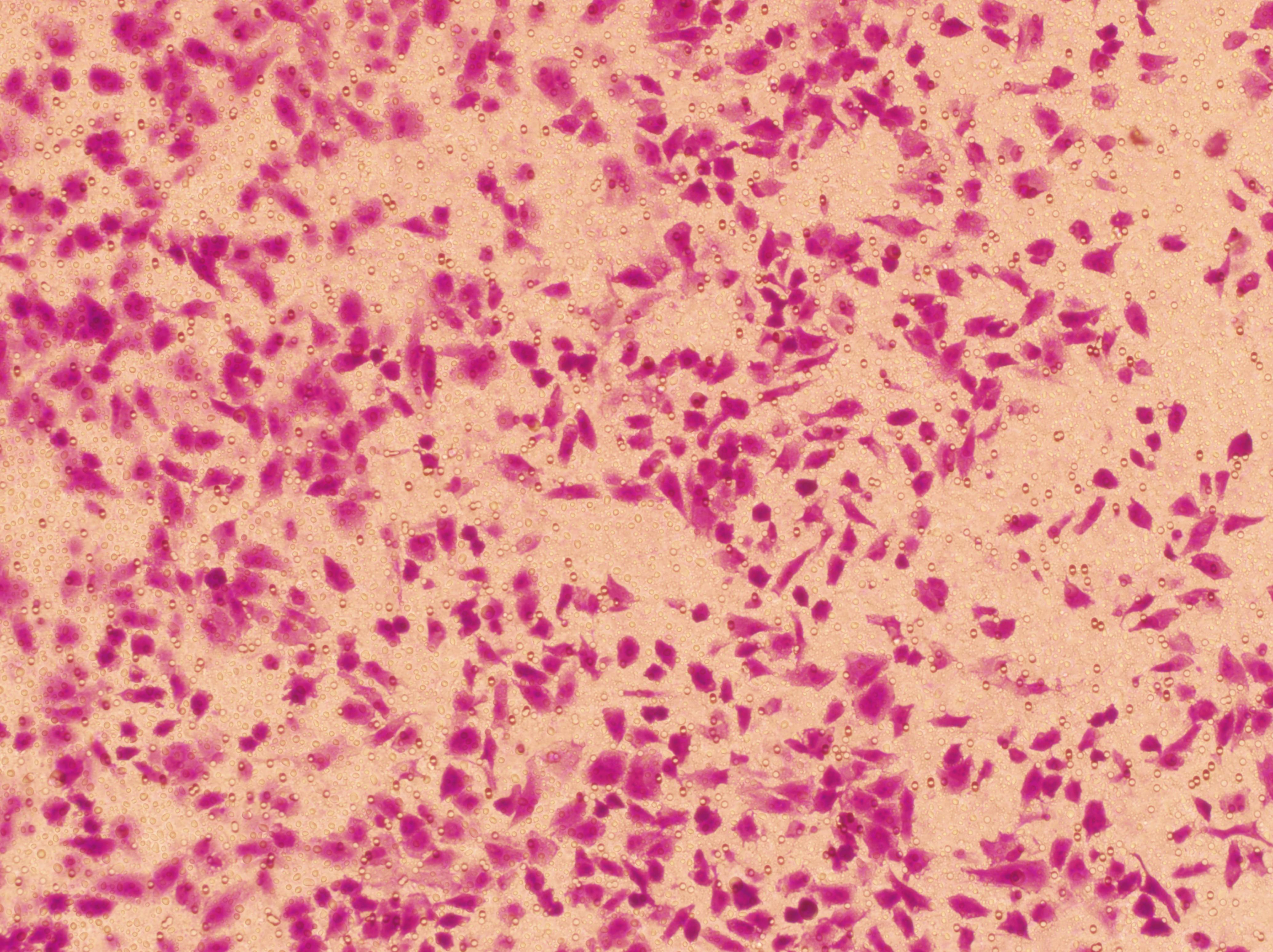

Supplement: Supplemental Information 12 — Migrated cells after silencing LINC01936 were photographed under IX71 inverted fluorescence microscope (magnification, ×100) [file peerj-11-16447-s012.zip › S11.2 Migration Si-LINC01936/H1703-siRNA/Migration siNC H1703.jpg]

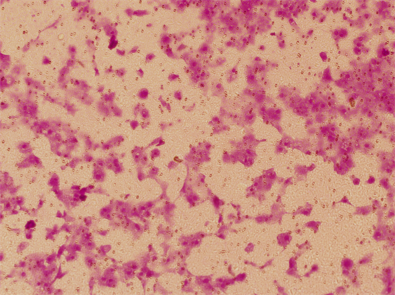

Supplement: Supplemental Information 12 — Migrated cells after silencing LINC01936 were photographed under IX71 inverted fluorescence microscope (magnification, ×100) [file peerj-11-16447-s012.zip › S11.2 Migration Si-LINC01936/KNS-62-siRNA/Migration siNC KNS-62.tif]

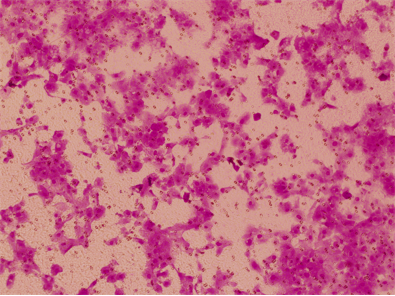

Supplement: Supplemental Information 12 — Migrated cells after silencing LINC01936 were photographed under IX71 inverted fluorescence microscope (magnification, ×100) [file peerj-11-16447-s012.zip › S11.2 Migration Si-LINC01936/KNS-62-siRNA/Migration siLINC01936 KNS-62.tif]

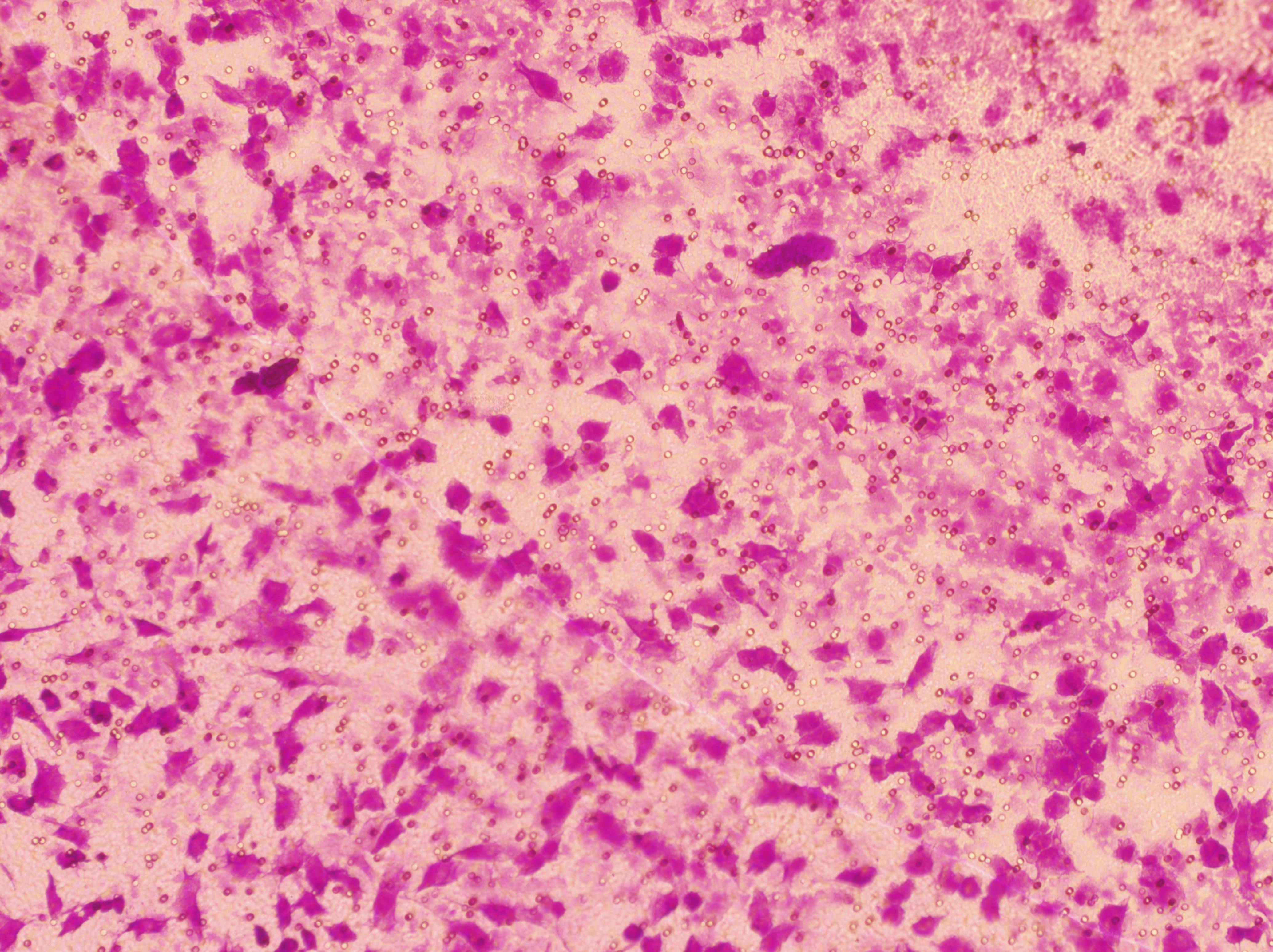

Supplement: Supplemental Information 14 — The migrated cells after silencing LINC01936 were photographed under IX71 inverted fluorescence microscope (magnification, ×200) [file peerj-11-16447-s014.zip › S12.2 Invasion Si-LINC01936/H1703-siRNA/Invasion siLINC01936 H1703.jpg]

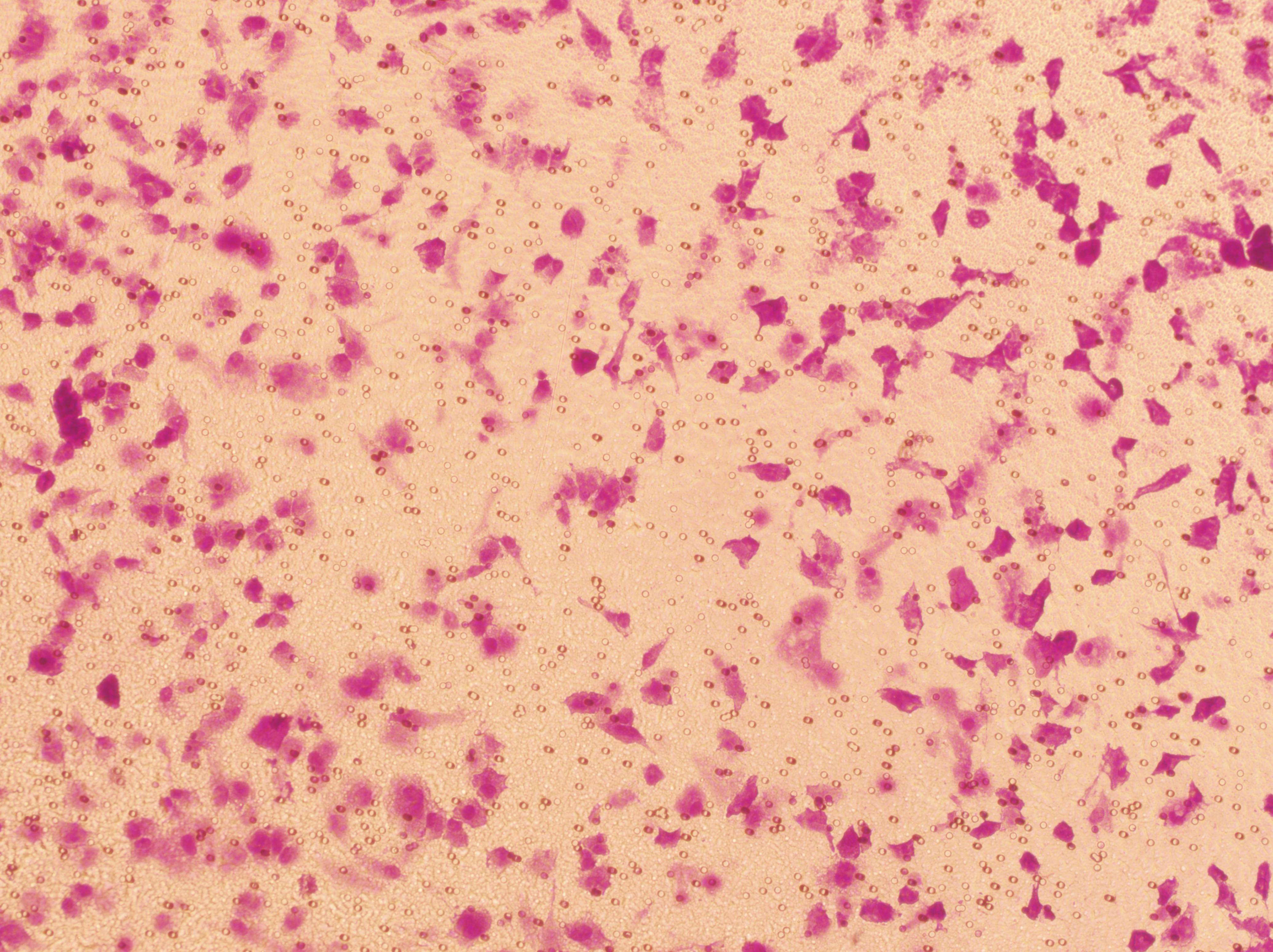

Supplement: Supplemental Information 14 — The migrated cells after silencing LINC01936 were photographed under IX71 inverted fluorescence microscope (magnification, ×200) [file peerj-11-16447-s014.zip › S12.2 Invasion Si-LINC01936/H1703-siRNA/Invasion siNC H1703.jpg]

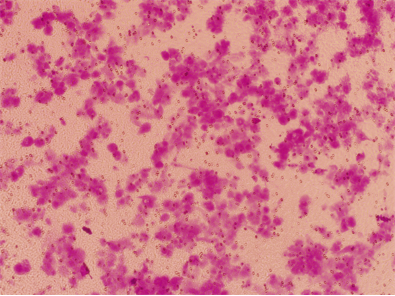

Supplement: Supplemental Information 14 — The migrated cells after silencing LINC01936 were photographed under IX71 inverted fluorescence microscope (magnification, ×200) [file peerj-11-16447-s014.zip › S12.2 Invasion Si-LINC01936/KNS-62-SiRNA/Invasion siLINC01936 KNS-62.tif]

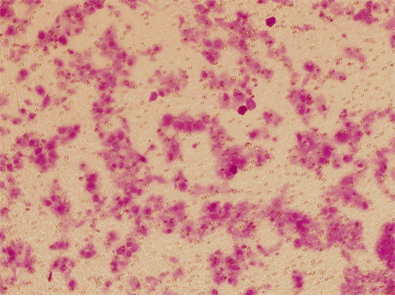

Supplement: Supplemental Information 14 — The migrated cells after silencing LINC01936 were photographed under IX71 inverted fluorescence microscope (magnification, ×200) [file peerj-11-16447-s014.zip › S12.2 Invasion Si-LINC01936/KNS-62-SiRNA/Invasion siNC KNS-62.tif]
