# Supplementary material for: LINC01936 inhibits the proliferation and metastasis of lung squamous cell carcinoma probably by EMT signaling and immune infiltration
Source: PeerJ. 2023 Dec 7;11:e16447. doi: 10.7717/peerj.16447 (PMC10710776; doi:10.7717/peerj.16447)
Supplement: Supplemental Information 2 — The differentially expressed lncRNAs from GSE88862 was identified by the online analytic tool GEO2R provided by the GEO database. [file peerj-11-16447-s002.zip › S2.GSE88862 differentially expressed genes/GSE88862-limma_package.zip!/GSE88862-limma-vol/volcano.pdf]

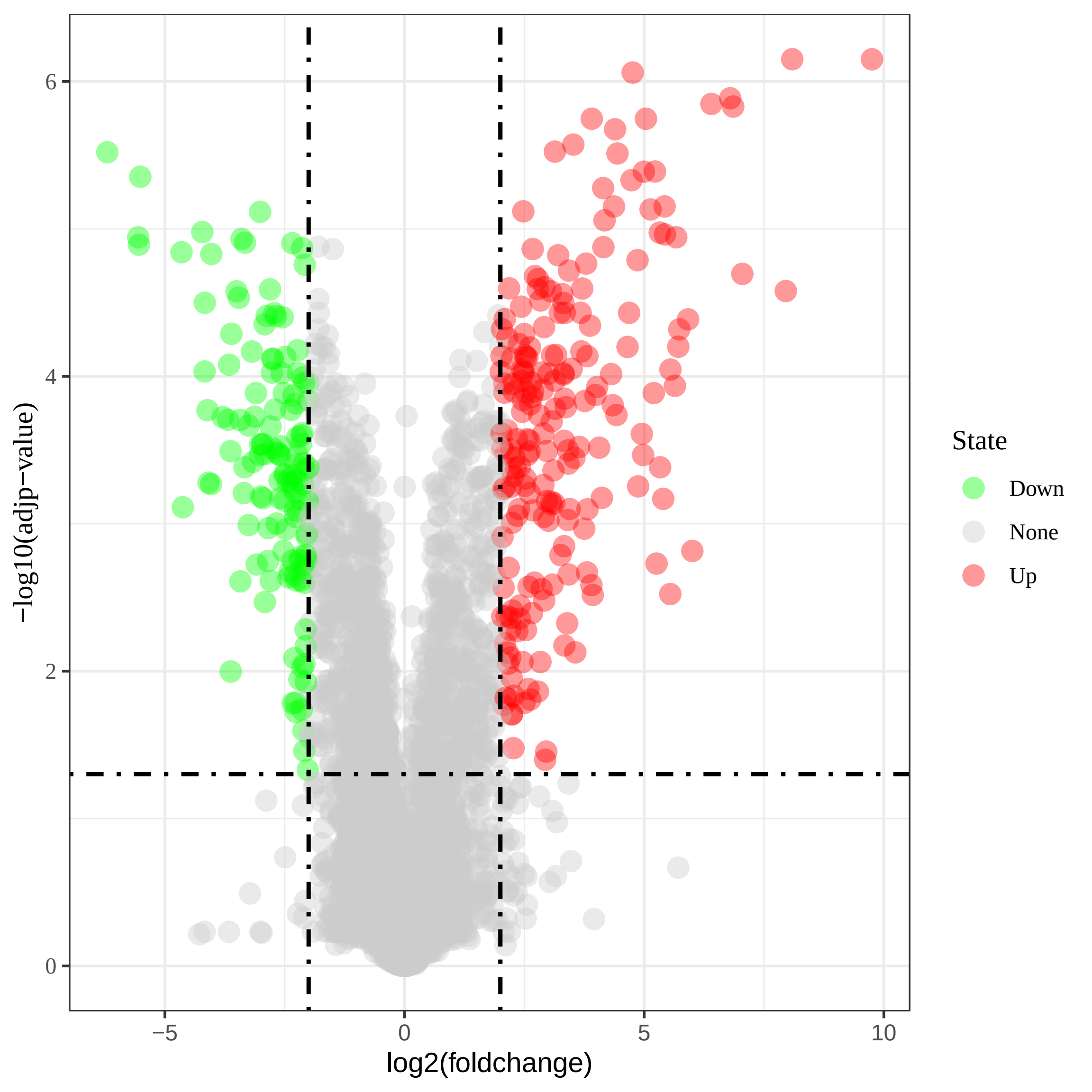

−log10(adjp−value)
6
4
2
0
−5
0
5
10
log2(foldchange)
State
Down
None
Up
